# Supplementary material for: Distinct Clinicopathological Features and Prognostic Values of High-, Low-, or Non-Expressing HER2 Status in Colorectal Cancer
Source: Cancers (Basel). 2023 Jan 16;15(2):554. doi: 10.3390/cancers15020554 (PMC9856362; doi:10.3390/cancers15020554)

Figure S1: Kaplan-Meier survival analysis for disease-free survival. Comparison of HER2-zero and HER2-high (a), HER2-low and HER2-high (b), HER2-zero and HER2-low (c) for cohorts after propensity score matching. p values are form the Cox proportional hazards regression model

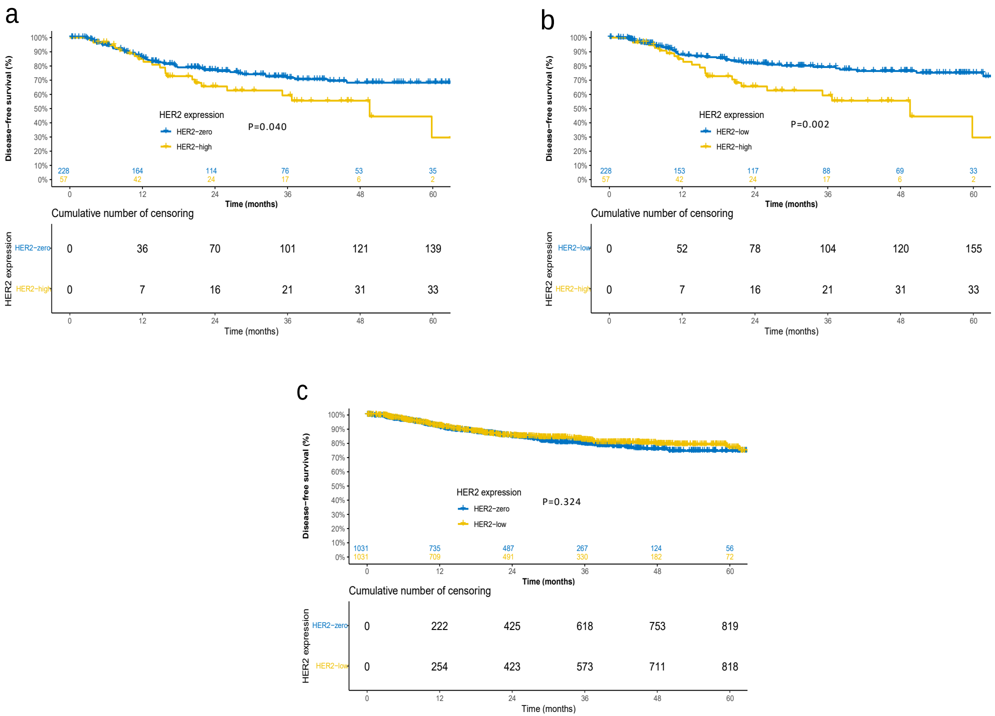

Supplement: Supplementary file 1 [file cancers-15-00554-s001.zip › Figure S1.pdf]
