# Supplementary material for: Distinct Clinicopathological Features and Prognostic Values of High-, Low-, or Non-Expressing HER2 Status in Colorectal Cancer
Source: Cancers (Basel). 2023 Jan 16;15(2):554. doi: 10.3390/cancers15020554 (PMC9856362; doi:10.3390/cancers15020554)
Supplement: Supplementary file 1 [file cancers-15-00554-s001.zip › Table S1.pdf]

Table S1. Baseline characteristics of the four groups of HER2 expression

Characteristics	Missing values	All the population, n=2768	HER2 IHC 0 group, n=1680	HER2 IHC 1 group, n=648	HER2 IHC 2 group, n=383	HER2 IHC 3 group, n=57	*P*
		No. (%)	No. (%)	No. (%)	No. (%)	No. (%)	
Age, years							
< 60		1325 (47.9%)	804 (47.9%)	310 (47.8%)	180 (47.0%)	31 (54.4%)	0.780
≧60		1443 (52.1%)	876 (52.1%)	338 (52.2%)	203 (53.0%)	26 (45.6%)	
Gender							
Female		1125 (40.6%)	695 (41.4%)	252 (38.9%)	154 (40.2%)	24 (42.1%)	0.735
Male		1643 (59.4%)	985 (58.6%)	396 (61.1%)	229 (59.8%)	33 (57.9%)	
Grade of differentiation							
Well- or moderately		2373 (85.7%)	1395 (83.0%)	578 (89.2%)	347 (90.6%)	53 (93.0%)	<0.001
Poorly		395 (14.3%)	285 (17.0%)	70 (10.8%)	36 (9.4%)	4 (7.0%)	
Primary tumor site							
Left (splenic flexure, descending colon, sigmoid colon, and rectum)		1685 (60.9%)	1010 (60.1%)	397 (61.3%)	241 (62.9%)	37 (64.9%)	0.680
Right (cecum, ascending colon, hepatic flexure, and transverse colon)		1083 (39.1%)	670 (39.9%)	251 (38.7%)	142 (37.1%)	20 (35.1%)	
Rectal cancer							
No		2719 (98.2%)	1652 (98.3%)	635 (98.0%)	377 (98.4%)	55 (96.5%)	0.707
Yes		49 (1.8%)	28 (1.7%)	13 (2.0%)	6 (1.6%)	2 (3.5%)	

Initial bowel obstruction						
No	2631 (95.1%)	1578 (93.9%)	626 (96.6%)	374 (97.7%)	53 (93.0%)	0.003
Yes	137 (4.9%)	102 (6.1%)	22 (3.4%)	9 (2.3%)	4 (7.0%)	
Vascular invasion and/or lymphatic infiltration						
No	2471 (89.3%)	1483 (88.3%)	594 (91.7%)	347 (90.6%)	47 (82.5%)	0.028
Yes	297 (10.7%)	197 (11.7%)	54 (8.3%)	36 (9.4%)	10 (17.5%)	
Perineural invasion						
No	2375 (85.8%)	1440 (85.7%)	563 (86.9%)	333 (86.9%)	39 (68.4%)	0.002
Yes	393 (14.2%)	240 (14.3%)	85 (13.1%)	50 (13.1%)	18 (31.6%)	
No. of lymph nodes excised						
< 12	284 (10.3%)	160 (9.5%)	70 (10.8%)	48 (12.5%)	6 (10.5%)	0.341
≧12	2484 (89.7%)	1520 (90.5%)	578 (89.2%)	335 (87.5%)	51 (89.5%)	
Pathologic T stage						
T1-T3	2349 (84.9%)	1467 (87.3%)	531 (81.9%)	304 (79.4%)	47 (82.5%)	<0.001
T4	419 (15.1%)	213 (12.7%)	117 (18.1%)	79 (20.6%)	10 (17.5%)	
Lymph node metastasis						
No	1770 (63.9%)	1064 (63.3%)	426 (65.7%)	252 (65.8%)	28 (49.1%)	0.066
Yes	998 (36.1%)	616 (36.7%)	222 (34.3%)	131 (34.2%)	29 (50.9%)	
Tumor deposit						
No	2283 (82.5%)	1394 (83.0%)	533 (82.3%)	315 (82.2%)	41 (71.9%)	0.194
Yes	485 (17.5%)	286 (17.0%)	115 (17.7%)	68 (17.8%)	16 (28.1%)	
Pathologic N stage						
N0	1622 (58.6%)	977 (58.2%)	393 (60.6%)	227 (59.3%)	25 (43.9%)	0.092
N1-2	1146 (41.4%)	703 (41.8%)	255 (39.4%)	156 (40.7%)	32 (56.1%)	

Mismatch repair status							
Proficient		2429 (87.8%)	1472 (87.6%)	566 (87.3%)	336 (87.7%)	55 (96.5%)	0.243
Deficient		339 (12.2%)	208 (12.4%)	82 (12.7%)	47 (12.3%)	2 (3.5%)	
RAS/RAF mutation							
No		854 (49.4%)	528 (47.9%)	176 (49.3%)	117 (51.3%)	33 (80.5%)	0.001
Yes		875 (50.6%)	575 (52.1%)	181 (50.7%)	111 (48.7%)	8 (19.5%)	
Missing values	1039						
Neoadjuvant therapy							
No		2765 (99.9%)	1677 (99.8%)	648 (100%)	383 (100%)	57 (100%)	0.584
Yes		3 (0.1%)	3 (0.2%)	0 (0%)	0 (0%)	0 (0%)	
Adjuvant therapy							
No		1377 (49.7%)	828 (49.3%)	332 (51.2%)	194 (50.7%)	23 (40.4%)	0.414
Yes		1391 (50.3%)	852 (50.7%)	316 (48.8%)	189 (49.3%)	34 (59.6%)	
