# Supplementary material for: Distinct Clinicopathological Features and Prognostic Values of High-, Low-, or Non-Expressing HER2 Status in Colorectal Cancer
Source: Cancers (Basel). 2023 Jan 16;15(2):554. doi: 10.3390/cancers15020554 (PMC9856362; doi:10.3390/cancers15020554)
Supplement: Supplementary file 1 [file cancers-15-00554-s001.zip › Table S2.pdf]

Table S2. Baseline characteristics of HER2 IHC score 1and HER2 IHC score 2

Characteristics	Missing values	HER2 IHC 1 group, n=648	HER2 IHC 2 group, n=383	*P*
		No. (%)	No. (%)	
Age, years				
< 60		310 (47.8%)	180 (47.0%)	0.844
≧60		338 (52.2%)	203 (53.0%)	
Gender				
Female		252 (38.9%)	154 (40.2%)	0.724
Male		396 (61.1%)	229 (59.8%)	
Grade of differentiation				
Well- or moderately		578 (89.2%)	347 (90.6%)	0.541
Poorly		70 (10.8%)	36 (9.4%)	
Primary tumor site				
Left (splenic flexure, descending colon, sigmoid colon, and rectum)		397 (61.3%)	241 (62.9%)	0.643
Right (cecum, ascending colon, hepatic flexure, and transverse colon)		251 (38.7%)	142 (37.1%)	
Rectal cancer				
No		635 (98.0%)	377 (98.4%)	0.789
Yes		13 (2.0%)	6 (1.6%)	
Initial bowel obstruction				
No		626 (96.6%)	374 (97.7%)	0.447
Yes		22 (3.4%)	9 (2.3%)	
Vascular invasion and/or lymphatic infiltration				
No		594 (91.7%)	347 (90.6%)	0.637
Yes		54 (8.3%)	36 (9.4%)	
Perineural invasion				
No		563 (86.9%)	333 (86.9%)	1.000
Yes		85 (13.1%)	50 (13.1%)	
No. of lymph nodes excised				
< 12		70 (10.8%)	48 (12.5%)	0.458
≧12		578 (89.2%)	335 (87.5%)	
Pathologic T stage				
T1-T3		531 (81.9%)	304 (79.4%)	0.350
T4		117 (18.1%)	79 (20.6%)	
Lymph node metastasis				

No		426 (65.7%)	252 (65.8%)	1.000
Yes		222 (34.3%)	131 (34.2%)	
Tumor deposit				
No		533 (82.3%)	315 (82.2%)	1.000
Yes		115 (17.7%)	68 (17.8%)	
Pathologic N stage				
N0		393 (60.6%)	227 (59.3%)	0.710
N1-2		255 (39.4%)	156 (40.7%)	
Mismatch repair status				
Proficient		566 (87.3%)	336 (87.7%)	0.935
Deficient		82 (12.7%)	47 (12.3%)	
RAS/RAF mutation				
No		176 (49.3%)	117 (51.3%)	0.696
Yes		181 (50.7%)	111 (48.7%)	
Missing values	446			
Neoadjuvant therapy				
No		648 (100%)	383 (100%)	NA
Yes		0 (0%)	0 (0%)	
Adjuvant therapy				
No		332 (51.2%)	194 (50.7%)	0.908
Yes		316 (48.8%)	189 (49.3%)	
