# Supplementary material for: Distinct Clinicopathological Features and Prognostic Values of High-, Low-, or Non-Expressing HER2 Status in Colorectal Cancer
Source: Cancers (Basel). 2023 Jan 16;15(2):554. doi: 10.3390/cancers15020554 (PMC9856362; doi:10.3390/cancers15020554)
Supplement: Supplementary file 1 [file cancers-15-00554-s001.zip › Table S3.pdf]

Table S3. Selected baseline characteristics before and after propensity score matching in HER2-zero and HER2-high group

**Characteristics**	**No. (%)**				**No. (%)**			
	**Before matching**				**After matching**			
	**HER2-zero group, n=1680**	**HER2-high group, n=57**	***P***	**Standardized difference**	**HER2-zero group, n=228**	**HER2-high group, n=57**	***P***	**Standardized difference**
Age, years								
< 60	804 (47.9%)	31 (54.4%)	0.403	0.130	123 (53.9%)	31 (54.4%)	1	0.009
≧60	876 (52.1%)	26 (45.6%)			105 (46.1%)	26 (45.6%)		
Initial bowel obstruction								
No	1578 (93.9%)	53 (93.0%)	0.990	0.038	213 (93.4%)	53 (93.0%)	1	0.017
Yes	102 (6.1%)	4 (7.0%)			15 (6.6%)	4 (7.0%)		
Grade of differentiation								
Well- or moderately	1395 (83.0%)	53 (93.0%)	0.072	0.309	217 (95.2%)	53 (93.0%)	0.74	0.093
Poorly	285 (17.0%)	4 (7.0%)			11 (4.8%)	4 (7.0%)		
Pathologic T stage								
T1-T3	1467 (87.3%)	47 (82.5%)	0.380	0.135	193 (84.6%)	47 (82.5%)	0.839	0.059
T4	213 (12.7%)	10 (17.5%)			35 (15.4%)	10 (17.5%)		
Vascular invasion and/or lymphatic infiltration								
No	1483 (88.3%)	47 (82.5%)	0.260	0.164	195 (85.5%)	47 (82.5%)	0.71	0.084
Yes	197 (11.7%)	10 (17.5%)			33 (14.5%)	10 (17.5%)		
Perineural invasion								
No	1440 (85.7%)	39 (68.4%)	0.001	0.418	147 (64.5%)	39 (68.4%)	0.686	0.084
Yes	240 (14.3%)	18 (31.6%)			81 (35.5%)	18 (31.6%)		

Mismatch repair status								
Proficient	1472 (87.6%)	55 (96.5%)	0.070	0.332	219 (96.1%)	55 (96.5%)	1	0.023
Deficient	208 (12.4%)	2 (3.5%)			9 (3.9%)	2 (3.5%)		
Lymph node metastasis								
No	1064 (63.3%)	28 (49.1%)	0.041	0.288	122 (53.5%)	28 (49.1%)	0.656	0.088
Yes	616 (36.7%)	29 (50.9%)			106 (46.5%)	29 (50.9%)		
Tumor deposit								
No	1394 (83.0%)	41 (71.9%)	0.047	0.265	180 (78.9%)	41 (71.9%)	0.338	0.164
Yes	286 (17.0%)	16 (28.1%)			48 (21.1%)	16 (28.1%)		
No. of lymph nodes excised								
< 12	160 (9.5%)	6 (10.5%)	0.981	0.033	205 (89.9%)	51 (89.5%)	1	0.014
≧12	1520 (90.5%)	51 (89.5%)			23 (10.1%)	6 (10.5%)		
Rectal cancer								
No	1652 (98.3%)	55 (96.5%)	0.594	0.116	221 (96.9%)	55 (96.5%)	0.866	0.024
Yes	28 (1.7%)	2 (3.5%)			7 (3.1%)	2 (3.5%)		
