# Supplementary material for: Distinct Clinicopathological Features and Prognostic Values of High-, Low-, or Non-Expressing HER2 Status in Colorectal Cancer
Source: Cancers (Basel). 2023 Jan 16;15(2):554. doi: 10.3390/cancers15020554 (PMC9856362; doi:10.3390/cancers15020554)
Supplement: Supplementary file 1 [file cancers-15-00554-s001.zip › Table S4.pdf]

Table S4. Selected baseline characteristics before and after propensity score matching in HER2-low and HER2-high group

Characteristics	No. (%)				No. (%)			
	**Before matching**				**After matching**			
	**HER2-low group, n=1031**	**HER2-high group, n=57**	***P***	**Standardized difference**	**HER2-low group, n=228**	**HER2-high group, n=57**	***P***	**Standardized difference**
Age, years								
< 60	490 (47.5%)	31 (54.4%)	0.383	0.137	125 (54.8%)	31 (54.4%)	1	0.009
≧60	541 (52.5%)	26 (45.6%)			103 (45.2%)	26 (45.6%)		
Initial bowel obstruction								
No	1000 (97.0%)	53 (93.0%)	0.199	0.183	209 (91.7%)	53 (93.0%)	0.957	0.049
Yes	31 (3.0%)	4 (7.0%)			19 (8.3%)	4 (7.0%)		
Grade of differentiation								
Well- or moderately	925 (89.7%)	53 (93.0%)	0.569	0.116	208 (91.2%)	53 (93.0%)	0.873	0.065
Poorly	106 (10.3%)	4 (7.0%)			20 (8.8%)	4 (7.0%)		
Pathologic T stage								
T1-T3	835 (81.0%)	47 (82.5%)	0.919	0.038	204 (89.5%)	47 (82.5%)	0.217	0.202
T4	196 (19.0%)	10 (17.5%)			24 (10.5%)	10 (17.5%)		
Vascular invasion and/or lymphatic infiltration								
No	941 (91.3%)	47 (82.5%)	0.045	0.262	199 (87.3%)	47 (82.5%)	0.464	0.134
Yes	90 (8.7%)	10 (17.5%)			29 (12.7%)	10 (17.5%)		
Perineural invasion								
No	896 (86.9%)	39 (68.4%)	<0.001	0.452	157 (68.9%)	39 (68.4%)	1	0.009
Yes	135 (13.1%)	18 (31.6%)			71 (31.1%)	18 (31.6%)		

Mismatch repair status								
Proficient	902 (87.5%)	55 (96.5%)	0.068	0.335	220 (96.5%)	55 (96.5%)	1	<0.001
Deficient	129 (12.5%)	2 (3.5%)			8 (3.5%)	2 (3.5%)		
Lymph node metastasis								
No	678 (65.8%)	28 (49.1%)	0.016	0.340	122 (53.5%)	28 (49.1%)	0.656	0.087
Yes	353 (34.2%)	29 (50.9%)			106 (46.5%)	29 (50.9%)		
Tumor deposit								
No	848 (82.3%)	41 (71.9%)	0.074	0.246	170 (74.6%)	41 (71.9%)	0.813	0.059
Yes	183 (17.7%)	16 (28.1%)			58 (25.4%)	16 (28.1%)		
No. of lymph nodes excised								
< 12	118 (11.4%)	6 (10.5%)	1.000	0.029	198 (86.8%)	51 (89.5%)	0.755	0.081
≧12	913 (88.6%)	51 (89.5%)			30 (13.2%)	6 (10.5%)		
Rectal cancer								
No	1012 (98.2%)	55 (96.5%)	0.693	0.103	218 (95.6%)	55 (96.5%)	0.769	0.045
Yes	19 (1.8%)	2 (3.5%)			10 (4.4%)	2 (3.5%)		
