# Supplementary material for: Distinct Clinicopathological Features and Prognostic Values of High-, Low-, or Non-Expressing HER2 Status in Colorectal Cancer
Source: Cancers (Basel). 2023 Jan 16;15(2):554. doi: 10.3390/cancers15020554 (PMC9856362; doi:10.3390/cancers15020554)
Supplement: Supplementary file 1 [file cancers-15-00554-s001.zip › Table S5.pdf]

Table S5. Selected baseline characteristics before and after propensity score matching in HER2-zero and HER2-low group

Characteristics	No. (%)				No. (%)			
	Before matching				After matching			
	HER2-zero group, n=1680	HER2-low group, n=1031	*P*	Standardized difference	HER2-zero group, n=1031	HER2-low group, n=1031	*P*	Standardized difference
Age, years								
< 60	804 (47.9%)	490 (47.5%)	0.898	0.007	493 (47.8%)	490 (47.5%)	0.930	0.006
≧60	876 (52.1%)	541 (52.5%)			538 (52.2%)	541 (52.5%)		
Initial bowel obstruction								
No	1578 (93.9%)	1000 (97.0%)	<0.001	0.148	1002 (97.2%)	1000 (97.0%)	0.896	0.012
Yes	102 (6.1%)	31 (3.0%)			29 (2.8%)	31 (3.0%)		
Grade of differentiation								
Well- or moderately	1395 (83.0%)	925 (89.7%)	<0.001	0.196	924 (89.6%)	925 (89.7%)	1.000	0.003
Poorly	285 (17.0%)	106 (10.3%)			107 (10.4%)	106 (10.3%)		
Pathologic T stage								
T1-T3	1467 (87.3%)	835 (81.0%)	<0.001	0.174	867 (84.1%)	835 (81.0%)	0.072	0.082
T4	213 (12.7%)	196 (19.0%)			164 (15.9%)	196 (19.0%)		
Vascular invasion and/or lymphatic infiltration								
No	1483 (88.3%)	941 (91.3%)	0.016	0.099	937 (90.9%)	941 (91.3%)	0.817	0.014
Yes	197 (11.7%)	90 (8.7%)			94 (9.1%)	90 (8.7%)		
Perineural invasion								
No	1440 (85.7%)	896 (86.9%)	0.415	0.035	894 (86.7%)	896 (86.9%)	0.948	0.006
Yes	240 (14.3%)	135 (13.1%)			137 (13.3%)	135 (13.1%)		

Mismatch repair status								
Proficient	1472 (87.6%)	902 (87.5%)	0.968	0.004	890 (86.3%)	902 (87.5%)	0.473	0.034
Deficient	208 (12.4%)	129 (12.5%)			141 (13.7%)	129 (12.5%)		
Lymph node metastasis								
No	1064 (63.3%)	678 (65.8%)	0.215	0.051	689 (66.8%)	678 (65.8%)	0.641	0.023
Yes	616 (36.7%)	353 (34.2%)			342 (33.2%)	353 (34.2%)		
Tumor deposit								
No	1394 (83.0%)	848 (82.3%)	0.665	0.019	862 (83.6%)	848 (82.3%)	0.447	0.036
Yes	286 (17.0%)	183 (17.7%)			169 (16.4%)	183 (17.7%)		
No. of lymph nodes excised								
< 12	160 (9.5%)	118 (11.4%)	0.125	0.063	912 (88.5%)	913 (88.6%)	1.000	0.003
≧12	1520 (90.5%)	913 (88.6%)			119 (11.5%)	118 (11.4%)		
Rectal cancer								
No	1652 (98.3%)	1012 (98.2%)	0.850	0.013	1015 (98.4%)	1012 (98.2%)	0.609	0.023
Yes	28 (1.7%)	19 (1.8%)			16 (1.6%)	19 (1.8%)		
